# “Nobody Wants to Talk About It, Especially in This Building”: A Qualitative Study of How People Living in Permanent Supportive Housing Approach End-Of-Life Care

**DOI:** 10.1177/00302228221114756

**Published:** 2022-07-10

**Authors:** Emma K. McCune, Megan R. Visser, Joshua Bamberger

**Affiliations:** 1 12224 University of California, San Francisco, School of Medicine, San Francisco, CA, USA; 2 Department of Social and Behavioral Sciences, 8785 University of California, San Francisco, CA, USA; 3 Department of Family and Community Medicine, 8785 University of California, San Francisco, CA, USA

**Keywords:** death, dying, palliative care, end-of-life care, homelessness, permanent supportive housing, qualitative research

## Abstract

Permanent supportive housing (PSH) is long-term affordable housing with onsite social services. End-of-life care (EOLC) involves a discussion about the type of medical care an individual hopes to receive at the end of their life. This qualitative study examines the goals, desires, and expectations for EOLC for people living in PSH. Semi-structured interviews were conducted with 17 formerly homeless residents in four PSH facilities in San Francisco, California and analyzed using the framework method. The interviews reveal how an individual’s experience with housing precarity and with the PSH setting shape their preferences and expectations for the end of life. While PSH residents value social support in their final days, social isolation in PSH serves as a barrier to receiving such support. Results from this work can inform policies and programs to support people living in PSH in achieving their desired death.

## Introduction

Permanent supportive housing (PSH) is long-term, affordable housing with on-site medical and social services. Permanent supportive housing is an effective public health intervention for chronic homelessness, particularly with helping people experiencing homelessness (PEH) achieve and maintain stable housing (Aubry et al., 2020). In a randomized controlled trial, PEH who received PSH versus usual care experienced higher rates of housing retention and outpatient mental health services, along with lower rates of psychiatric emergency department and shelter use (Raven et al., 2020). A history of homelessness is associated with increased rates of long-term chronic illness and increased mortality when compared to the general population. Due to the health-related risks of chronic homelessness, people living in PSH are more likely to suffer from long-term chronic illness and have an increased risk of mortality compared to the general population (Feodor Nilsson et al., 2018; Sutherland et al., 2021). Little is known, however, about how people age and die in PSH (Henwood et al., 2015).

End-of-life care (EOLC) includes the opportunity for a dying person to discuss their wishes and desires for the type of medical care they will receive so that they can achieve their desired death (Ruland and Moore, 1998). People experiencing homelessness have described care needs and desires at the end of their lives, including nonjudgmental treatment with respect and dignity; social contact from family, friends, or social support network; and respect for their wishes in their final days via advance care planning and proxy decision-makers (Klop et al., 2018). However, interpersonal and institutional factors make it difficult for PEH to access palliative services at the end of their lives. The chaotic lifestyle often associated with being homeless and attitudes to healthcare systems (e.g. mistrust of healthcare professionals due to previous negative experiences, complex care needs and competing priorities) may prevent PEH from engaging in EOLC conversations with providers (Hudson et al., 2016). Psychosocial factors, such as family estrangement and substance use, make PEH difficult candidates for traditional models of palliative care and hospice services (Hudson et al., 2016). Mainstream healthcare systems have failed to provide adequate palliative care to PEH: healthcare professionals may not have the appropriate training or experience to provide palliative care to PEH, resulting in an environment in which PEH do not feel comfortable and accepted (Hudson et al., 2016; Klop et al., 2018) Suggested interventions to improve EOLC in this population include support homes, shelter-based palliative care, and coordination with harm-reduction services (Sumalinog et al., 2017).

Studies of EOLC issues among PEH explore how frequent experiences with death and loss shape a person’s perceptions of their own terminal illness and EOLC (West et al., 2020). In previous qualitative studies examining palliative care in the homeless population, PEH commonly described instances of witnessing the deaths of multiple peers secondary to drug use, violence, hunger, and cold exposure (Hudson et al, 2016). These observations of bad and lonely deaths created a fear of death and dying, particularly a fear of dying anonymously and alone (Klop et al., 2018). As a result, PEH often avoid thinking or talking about death and dying, and instead focus on the daily challenges of life (Hudson et al, 2016).

To date, little is known about whether the transition from homelessness to PSH, which includes permanent housing and a community of neighbors and staff, may change perceptions of EOLC. The aim of our qualitative study is to examine the goals, desires, and expectations for EOLC for people living in PSH in San Francisco, California. Findings of this study can inform policies and programs to support people living in PSH in achieving their desired death.

## Methods

### Design

Given that so little is known about the goals, desires and expectations for EOLC among PSH residents, our study aimed to shed light on the lived experiences of people living in this setting. We used a qualitative descriptive research methodology to explore end-of-life care and wishes among people living in a PSH setting (Willis et al., 2016). We conducted semi-structured interviews to solicit information about participants’ lives, their understanding of end-of-life issues, experiences with illness or death, and suggestions for improving EOLC in PSH. Qualitative descriptive research is rooted in naturalistic inquiry, which allowed us to collect and summarize responses from our participants, without requiring a pre-existing theory related to our topic. The goal of our design was to develop a thematic analysis guided by the commonalities and differences among the ideas from the participants in our study (Gale et al., 2013).

### Setting

This study was carried out in four supportive housing facilities in San Francisco, California that have on-site public health department clinical staff.

### Participant Recruitment

The Institutional Review Board approved this study and all participants provided written consent. Purposive sampling was used (Creswell and Poth, 2016). Individuals eligible to participate in the study were English-speaking, 18 years or older, and currently residing in PSH. On-site public health department staff members, including nurse practitioners and social workers, from the four PSH sites identified and approached eligible residents for recruitment. Permanent supportive housing staff members assisted in recruitment, because they knew the residents personally and were able to refer PSH residents who they believed would be able to speak to the topic of the interview. When a resident expressed interest in participation, the PSH staff coordinated with the study team to arrange an interview. The study team intentionally sought to engage any resident willing and able to participate, regardless of age, demographics, or state of health. As such, maximum variation sampling was not attempted. With a recruitment goal of 20 participants (five from each site), 17 individuals provided written consent and agreed to digital-taping and transcription of their interview. Each participant was paid a small stipend to compensate them for their time and was also made aware that they had the right to withdraw from the study at any time.

## Data Collection

Between July 15–30, 2020, all interviews were conducted in-person at the PSH where the participants lived. Each interview lasted approximately 45 minutes (ranging from 13 to 67 minutes), and was audio-recorded, transcribed using Production Transcripts, and checked for accuracy by investigator E.M. Consistent with the qualitative descriptive method, a semi-structured interview schedule of 22 questions was used (Table 1). Open-ended interview questions created a disclosive space in which the participant was invited to share with the interviewer their personal experiences and desires for end-of-life care within the context of their life history and daily life in PSH. The interview guide centered around four major topic areas, including life; illness and injury; death and dying; and end-of-life care. Early questions explored the resident’s history with housing, particularly how they ended up in PSH, and their experiences with illness and injury, both personal and observed through others. Later questions explored the resident’s perceptions of death and dying and solicited their perspectives of end-of-life care in PSH.

**Table 1.** Semi-Structured Interview Guide.

Theme	Questions
Life	• How long have you lived in the building?
	• Describe your housing situation in the past.
	• How is your health overall?
Illness/Injury	• What experience have you had with serious illness or injury?
	o How have your experiences with illness/injury impacted your life?
	• What experience have you had with a close friend or relative who had a serious illness or injury or who has died?
	o How did that person’s death affect how you think about your own life, or the type of care you would want if you got very sick?
Death and Dying	• We’re all going to die. When that time comes, and we hope it’s a long time from now, what would be the best situation for you?
	• If you had a choice, where would you be when you took your last breaths? Who would be with you?
	• Describe what a “good death” would look like for you
	o Are there things that you hope will happen when you die?
	o Are there things that you do not want to happen when you die?
	• What do you think might stand in the way of you having a good death?
	• What type of things would be most important to you in your final days?
	• Is reconnecting with friends or family at the end of life an important thing for you?
	o If so, who would you want to reconnect with?
	• Are you worried about what will happen to your body or belongings after you die?
	• What concerns do you have about having enough support, or having people there for you when you need them?
	• What worries you most when you think about your last days?
	• Who in your life could you talk to about these issues?
	o Have you ever had a conversation with [person] about how you feel about these things? If not, what are the barriers to having that conversation?
	• How prepared do you feel to have these conversations with people taking care of you, such as medical providers, staff, family, friends?
	• If not, what are barriers to having that conversation?
	• Who in your life would you trust to speak for you, if you weren’t able to tell people what you wanted? If you needed extra help?
End of Life care in PSH	• What experience have you had with neighbors or others in your building getting very sick or dying?
	o How was this news shared with you? How did it make you feel?
	o How do those experiences affect how you think about your own life, or the type of care you would want if you got very sick?
	• What concerns do you have about other people in your building spending their last days at home?
	• What type of things (services, people, support) would help more people in your building to live well in their last days at home?
	• What concerns would you have about dying at home or the end-of-life care you would get at home?

## Data Analysis

Inductive analysis of the data was performed using the framework method, which provided a clear structure for developing themes from the participant responses in the interview data (Heath et al., 2012; Gale et al., 2013). Widely used in health research, the framework method’s approach located commonalities and differences within qualitative data, created a working set of descriptive labels (codes) with which to analyze all the data, and then, obtained a holistic overview of the entire data set. The framework method’s hallmark is the summary of data in the form of a matrix, in order to analyze the output by individual case (participant) and by code (descriptive label assigned to raw excerpts). The framework method was appropriate for our research objectives because we were able to describe the themes related to our research questions about death, dying, and end-of-life care in PSH, as well as connect the views of each PSH resident participant to other aspects of their interview. This structure offered the flexibility to consider unforeseen responses of participants and relevant features of participants’ social context. Also, the framework method was shown to be an effective analytical process for research teams of two or more investigators, in which researchers brought different disciplinary perspectives and levels of familiarity with qualitative research. Throughout analysis, we documented our procedure in detail, aligning it with the methodological structure (Gale et al., 2013).

*Stage 1: Transcription.* Verbatim transcripts of each recorded interview were formatted for later coding and reviewed for accuracy by the interviewer (E.M.).

*Stage 2: Familiarization with the interviews.* Two investigators (E.M. and M.V.) independently reviewed interview transcripts to immerse themselves in the data and introduce M.V. to the context and setting of the interviews. Marginal notes also included analytical ideas and other first impressions were documented.

*Stage 3: Coding.* E.M. and M.V. independently reviewed three transcripts line-by-line, applying descriptive labels (“codes”) to raw excerpts of data to generate classifications of all data so that it can later be compared systematically. “Open-coding” alerted them to unexpected codes or passages that first appeared to not “fit” with the rest of the data; this holistic process began to develop emerging themes and potential challenges to dominant perspectives, which would become important during later steps in the analysis. Interviews were coded using Dedoose, version 8.3.40.

*Stage 4: Developing and applying a working analytical framework.* E.M. and M.V. met to compare their individual processes in order to ensure consistency between the team members. Together, they developed and refined a preliminary codebook of codes, which could systematically be applied to all transcripts. The codebook helped to shape a working analytical framework composed of categories of codes that the team clearly defined. E.M. and M.V. repeated the coding process with all transcripts, based on the working analytical framework, until no new themes emerged.

*Stage 5: Charting data into the framework matrix.* The data from each transcript were charted into a framework matrix to summarize the data by category. E.M. and M.V. included references to important quotations from the interviews that provided strong illustrations of codes or categories. They met to compare styles of charting and ensure consistency in their approach.

*Stage 6: Interpreting the data.* E.M. and M.V. reviewed and interpreted the framework matrix, individually writing analytical memos to make connections between codes, within individual cases and across the sample. Analysis meetings included discussion of recurring themes and deviant cases, as well as individual researcher’s reflexive and critical engagement with participant responses. Between meetings, each investigator wrote up their findings and developed rich descriptions of themes in further memos, which were then shared with the other investigator. Individually, they re-read each recurring theme description and checked the matrix as to whether there was adequate evidence for each theme. Final themes were derived collaboratively by E.M. and M.V. in subsequent analysis meetings (Gale et al., 2013).

### Rigor

The qualitative description design used in this study facilitated a process of ensuring rigor in the methods, analysis, and presentation of our findings. The authors documented the collection and analysis of the data to ensure the procedures continued to fit the overall goals of the study at each step. In particular, the framework method’s systematic structure provided us with multiple opportunities to account for the credibility of the data collected from interviews and the trustworthiness of our interpretations. The first author, who served as the interviewer, reviewed all transcripts for accuracy prior to coding the data, and kept detailed notes of her interactions. The second author, highly trained in qualitative research, also shared in each stage of the analysis process. Because very few studies have examined end-of-life care among PSH residents, our study required us to derive many possible coding categories from the data in this area, prior to developing themes or generating an interpretation. With this in mind, E.M. and M.V. reviewed transcripts independently, before constructing a codebook and the framework matrix. Thus, in shaping the codebook, we were able to render the common perspectives shared by participants, as well as diverse and contradictory ideas about the end-of-life. The iterative coding and memo-writing process allowed the team to resolve differences between coders and reflect together about the depth and logic of their interpretations. The framework matrix was revised in multiple stages as in-depth discussions of the data helped to ground the analysis more firmly in the data. Use of software for coding and organizing data allowed us to reliably develop a rich description of themes by selecting illustrative quotes across the sample and within cases. By presenting our results and discussing them in the context of relevant literature on people experiencing homelessness and end-of-life care, we provided further justification of our findings.

## Results

Seventeen participants were interviewed in four supportive housing sites in San Francisco, California. Study population demographics are summarized in Table 2: the average age of participants was 65 years, 52% of participants identified as male, and 47% were African American/Black. Individual level information is not included to ensure participant anonymity. These interviews highlighted four overarching themes regarding the end of life: (1) a lifetime of housing precarity shapes preferences; (2) social isolation extends into PSH; (3) the presence of other people makes a difference; (4) PSH staff provides emotional and practical support. Overall, the interviews revealed a dissonance between how a person living in PSH would prefer to spend their final days and how they expect to spend their final days. While residents identified the positive impact of exiting chronic homelessness through PSH, they also described structural and interpersonal challenges to achieving a desirable death, ones that are embedded in the PSH system itself. They also identified ways in which the PSH community could provide support during the dying process.

**Table 2.** Demographics of Study Participants.

Total Participants	17
Age (range)	65.5 (46–77)
Gender	
Male	9
Female	8
Race	
African American/Black	8
American Indian or Alaskan Native	2
Asian	2
Native Hawaiian or other pacific Islander	0
White/Caucasian	5

### A Lifetime of Housing Precarity Shapes Preferences for the End of Life

Residents reported preferences for the end of life related to location, final meals, and the afterlife that are shaped by a lifetime of housing precarity. All residents we interviewed said they would like to spend their final days in the PSH, in the hospital, at a private home, or in a hospice program, and many expressed a preference to die in a bed of their own. One resident asked for “a place of my own where I can walk around in” (Resident 5). Some preferred to die in a hospital: “Well, I probably am sick or injured, so I hope I’m in a comfortable hospital bed somewhere in a good hospital, maybe with a window with a tree outside” (Resident 10).

When asked about what they would want in their final days, 10 of the 17 residents were unable to directly answer the question. Some responded with uncertainty: “I can’t really imagine” (Resident 3). Others deflected: “I don’t know, because I do not think about it, not too much, and I don’t know…that’s a good question. I barely think about it right now, because I don’t want to” (Resident 12). Still others chose to “live day by day” and to “go on living,” rather than to plan and prepare for their final days. A number acquiesced to a lack of control over the outcome: “Death comes to us when it’s time. And you ain’t got no say in the matter. When it’s your time, it’s your time” (Resident 17).

Most participants were unaware of opportunities to receive help in end-of-life planning and hesitant to name preferences for the end of their lives during interviews. When options for EOLC (e.g., advance care directives or receiving care from others in PSH) were introduced in the interviews, residents described needing further assistance to plan for their deaths: “I need guidance. Someone to help me get, you know, do what I need to do” (Resident 13). Another resident, when asked who he could trust to care for and make decisions for him at the end of his life, said: “I’m going to wait until that happens, because if I were to choose one or two of the people here, they may be gone by the time I pass away” (Resident 10).

Several participants wished to be near loved ones when they are dying but felt that geographic distance or long-term estrangement would continue to prevent them from re-establishing a close connection. One woman wanted to be with her sister who lives in another state, however acknowledged that she could not do that because “I don’t want to be where she is and [she] where I am” and that, if she were to move in with her sister, “we’ll undoubtedly get into a fight and she’ll abandon me” (Resident 7). Some residents are estranged from their family: “I do have two brothers and two sisters, but…last time I saw any of them was back in ’85” (Resident 17). For a few residents, their family and loved ones have already passed: “my father died next to me when I was young…my mama passed away, and left me with my auntie, and then, suddenly, she passed away. So, I’m by myself. All my other people are gone, dead and gone” (Resident 14). Other residents stated that they would not want their family with them in their final days, citing challenging relationships as the reason: “my mom and I we don’t get along so I definitely don’t want her there” (Resident 2).

### Social isolation extends into PSH

Participants revealed experiences of social isolation living in PSH. Given the limitations created by PSH rules, some acknowledged that the amount of human contact they would want in their final days may not be feasible. As one woman said: “it’s just us in the room because we can’t have nobody else. They can visit but that’s about it. If you wanted to live with somebody, you would have to move somewhere else” (Resident 5). Another expected to be alone as they die: “I know ain’t nobody going to be around me. I’m just alone, by myself” (Resident 14). Some residents described a lack of close relationships with neighbors in PSH, stating that they “don’t get to know” or “don’t get along with my neighbors” because “I just usually stay in my room” or “I don’t normally talk to them.”

Concerns of social isolation at the end of life extend beyond the PSH setting and apply to other settings where residents may spend their final days. Though hospitals, hospices, and nursing facilities may offer more care resources than PSH, relocation to them at the end of life can also isolate PSH residents from their everyday support system. Participants noticed the isolation their neighbors and loved ones experienced in other settings. Resident 5 expressed remorse that her husband “died [in the hospital] and I wasn’t there” while another reflected upon her neighbor’s final days in a skilled nursing home: “He was isolated and that bothered me, the idea of being totally isolated…You know, getting older you’re isolated enough. You don’t need another layer of insulation on that isolation” (Resident 4).

Some residents felt like they could not discuss EOLC with PSH neighbors and staff. When asked who he could talk to about death and dying in PSH, one man said: “Nobody wants to talk about it, especially in this building. No, they just want to talk about the day-to-day activities of life” (Resident 10). Another woman shared that she had wanted to delegate another PSH resident as the power of attorney on her advance directive, but that “she and I kind of fell out, so I never really had the chance to continue the discussion with anyone” (Resident 2). Others identified challenges in serving as caretakers for their neighbors. When one of his neighbors was dying at a skilled nursing facility, “they asked a whole bunch of people here in the building if they would help out. Nope, didn’t want anything to do with it, and partly because it meant then that you have to go visit them…I ended up seeing him 36 times. That’s challenging, and without a car, it’s kind of expensive and tedious and tiring” (Resident 10).

Some described interpersonal challenges as barriers to receiving support from staff. When asked about what stood in the way of discussing death and dying with PSH staff, Resident 4 described her “strained relationship” with her social worker while several other participants believed the staff would not be interested in discussing EOLC with them. According to one woman, PSH residents do not know how to ask the staff for support in their final days. High staff turnover or staff burn-out may also be a barrier to PSH residents receiving adequate or timely support for planning and making decisions about their EOLC. One participant said: “I have yet to get mine [wishes] down on paper. The person I was working with to do that left, so it got left up in the air” (Resident 4). This inconsistent access to peer or staff support means that PSH residents are unable to communicate their EOLC preferences to others. Unfortunately, a few participants could not name a single trustworthy person in their lives to make decisions on their behalf, should they lose their decision-making capacity at the end of life.

### The Presence of Other People Makes A Difference

Discussions of social isolation experienced in PSH revealed the desire not to be alone at the end of life. Residents placed a high value on social support in their final days and expressed wishes to be near others as they approach death. When they were asked about their own final days, some participants expressed the desire to have someone to “check in” with them and/or to be joined by loved ones. Several described wanting visitors “see how I’m doing” or to see if they had died already. One participant asked for someone there, at least on occasion, so they “[don’t] feel alone or...frightened of ‘the change’” (Resident 4). Another participant felt comforted to have “someone who cares about me and who can help me get better, if I’m feeling sick or whatever, help me get better to help me get back on my feet” (Resident 16). One resident described the difference that the presence of another person would make in their final days: “If they ain’t got nobody there with them, that’s a bad thing. They knowing they going to die, I would rather be somewhere where some people are there or where somebody’s around…to take care of them or just comfort them and stuff like that” (Resident 9).

A few residents described the ability to confide in their friends living with them in PSH. One participant said her weekly hangout with two of her friends in PSH was “the only place where I’ve ever talked about, you know, this kind of stuff [death and dying]. And like the [advance] directive, and should you do an autopsy, should you not do an autopsy and different stuff like that” (Resident 1).

### PSH Staff Provides Emotional and Practical Support

The PSH environment played a strong role, both positively and negatively, in shaping residents’ perceptions of living in PSH at the end of their lives. On the one hand, PSH residents described supportive relationships with the staff and fellow residents. Yet, some PSH residents shared fears about dying alone and anonymously in their apartments. Interviews revealed that residents hoped to receive support from the PSH environment and community in their final days.

Many participants expressed fears about a delay in being found dead in their apartments. One resident, after observing how neighbors had died alone in their units, described PSH as a place “where they put you until you die” (Resident 13). One participant discussed her fear of dying alone in her apartment, after one of her neighbors “fell in his back room and hit his head on [the floor] and bled out” (Resident 4). They described instances when their neighbors were discovered dead in their units because “they don’t do wellness checks” or “because maggots started emerging from the room” (Resident 16, Resident 2). One woman hoped that “someone will get to me before I decompose because I don’t want that problem” (Resident 2). Struck by the number of people observed being wheeled out of rooms shrouded on gurneys after being found dead in their rooms, another participant strongly preferred to go to a hospital if they become very sick: “Put me in a hospital. Let me go there. Don’t let me go up in here. I’ve seen too many white sheets, okay?” (Resident 9). These observations and concerns highlighted the importance of having support and supervision throughout the dying process that may not always be assured in PSH. One resident stated that “over the weekend would be the hardest part for someone [to die]” because PSH staff were not working over the weekend (Resident 1). The residents who wished to be in a hospital when they die described the benefit of providers or others checking in on them several times a day during the dying process, noting that routine visits by PSH staff or other residents could not be guaranteed even once per day.

PSH residents expressed desires for the involvement of PSH staff, especially nurses and social workers, in providing emotional and practical support at the end of their lives. When asked about who in their lives could support them in their desired death, some participants identified PSH staff as the people they trusted most to be there and to make decisions for them. For Resident 2, PSH staff served this role “because I don’t have anyone else that I would confide in.” Resident 8 pulled his social worker aside during the interview to ask her if she could fill out an advance directive with him. She was the person he trusted most to ensure that his medical wishes were followed at the end of his life: “That’s what she would do. She’d be the one. Nobody else,” he said. One resident noticed that staff “being around, being available” helped other residents in their final days: “I know there have been people who’ve been ill, and [the social worker] goes, and she checks on them and helps out” (Resident 1). They felt confident that PSH staff would do all they could to make sure they were comfortable: “the staff would do whatever they could possibly do, you know, at the time” (Resident 1).

Participants also highlighted ways in which PSH could provide support in their final days by fulfilling basic needs and ensuring comfort. Some suggested, for instance, that PSH could offer additional support for its dying residents, having “someone with medical training” there to provide basic care in their final days. Participants suggested that support could be provided to help “if I had to go to the restroom or if I can’t get to the bathroom...clean me up,” or to be there to “make sure they have some entertainment and that it’s still functioning, make sure they have clean sheets on their bed, make sure the bathroom’s cleaned out really well” (Resident 16, Resident 10). One woman asked for “a nice, big, large-screen TV and a working telephone” so that she could “look at my DVDs and listen to my music.” One resident requested: “make sure I’m full. Make sure I done had a good meal” (Resident 2). Another asked for “something good to eat like ice cream or hot dogs” (Resident 10).

## Discussion

Though PSH residents have different life circumstances from people currently experiencing homelessness, most still carry the histories and lived experiences of chronic homelessness that inform their goals and expectations for their final days. This study demonstrates the importance for clinicians, staff, and policymakers to consult with those living in PSH about their EOLC wishes and expectations for support. Residents who participated in our study shared their thoughts of dying and social support at the end of life, including their perceptions of structural barriers and social challenges to achieving a desired death in PSH. They helped us to understand the unique features regarding EOLC in the transition from homelessness to housing and also made suggestions for improvement of EOLC in PSH to better support residents as they age. For instance, providers and caretakers could leverage existing relationships within PSH to support residents as they age, proactively introduce the discussion of death and dying, and provide emotional and social support at the end of life.

Our results expand upon previous research that explored the EOLC preferences of people experiencing a lifetime of housing precarity (West et al., 2020). Prior research has illustrated concerns that contribute to a “bad death” in the older homeless population, such as fears of a prolonged painful death, a sudden and violent death, anonymity upon death, or disrespect for the dying person (Wilson and Hewitt, 2018; Ko et al., 2015). In our study, we found similar concerns about death shared by PSH residents: they fear dying alone and anonymously in their rooms. For PEH and PSH residents, these fears and expectations for EOLC are shaped by experiences with illness and observations of the deaths of others (Dzul-Church et al., 2010; Håkanson et al., 2016; Song, Bartels, et al., 2007, Song, Ratner, et al., 2007; Tarzian et al., 2005; Tobey et al., 2017).

Permanent supportive housing as a context has unique attributes that distinguish it from chronic homelessness, transitional housing, or inpatient hospice care. This type of housing and the community within offer opportunities for social support at the end of life that improve upon the social conditions of PEH and influence how a person imagines a desirable dying process. Permanent supportive housing residents may face issues of social isolation highlighting the continuation of problems they experienced when they were unhoused or structural barriers in PSH that weaken relationships with family members or close friends outside of PSH (Hakanson et al., 2016). However, these very same residents may have the opportunity to form trusting relationships with the staff of residential programs (Dzul-Church et al., 2010; McNeil et al., 2013; Podymow et al., 2006). Permanent supportive housing residents in our study shared stories about the care that they received or offered to other residents as well as stories about care from staff or neighbors that they observed others receive at the end of their lives.

Many residents wished that PSH staff members would take a more active role as their surrogate decision-makers, offering medical and social support at the end of life. While these relationships can be leveraged to enhance social support during the dying process, it must be done so cautiously. If PSH staff are not trained to provide medical care to a dying person, they may be unprepared to attend to the attendant health and social conditions that arise at the end of life (Henwood et al., 2015; Hudson et. al, 2016). Likewise, if PSH staff are not trained to talk with comfort and knowledge about death and dying, residents might well have palliative needs that are unmet at the end of their lives (Krakowsky et al., 2013). To avoid PSH staff burnout and to meet the needs of residents, supportive housing programs should build staff capacity for EOLC decision-making with all PSH residents, not only those who are dying.

Previous research has suggested that models of advance care planning (ACP), such as relationship-centered care, integration of ACP into routine care, and documentation of ACP, may support PSH residents in exploring EOLC decision-making (Tarzian et al., 2005; Song et al., 2007; Kaplan-Weisman et al., 2019). Although several of our participants had filled out or were planning to fill out an advance directive, our interviews revealed a general lack of knowledge about the options surrounding EOLC and a paucity of discussion about ACP and surrogate decision-making. It would seem appropriate for the PSH community to initiate these ACP conversations and to inform residents of their EOLC options, providing a safer, more consistent setting for EOLC that is not available to the unhoused person.

### Strengths and Limitations

To our knowledge, this is the first qualitative study to elicit and examine EOLC preferences and expectations from PSH residents themselves. We believe that these voices and perspectives are valuable additions to the ongoing conversation about how to best meet the palliative needs of this population. The generalizability of our findings is limited by a small sample size study and the exclusion of non-English speaking residents. The recruitment methods may have also introduced selection bias, as on-site PSH staff who were asked to recruit participants may have approached PSH residents with whom they have positive or strong relationships. It is worth noting that we intentionally included participants regardless of age or state of health, instead of recruiting individuals who were ill or nearing the end of life.

## Conclusions

Our findings underscore the fact that PSH residents are capable decision-makers about their end-of-life care and aware of the existing limitations and advantages of their setting in receiving support at the end of life. Permanent supportive housing residents deserve the same respect for their wishes at the end of life as do those who have not experienced chronic homelessness. Dormitory-style housing of people nearing the end of their lives combined with inadequate access to palliative care or routine social services creates a unique public health problem. While it is true that PSH offers people who experienced chronic homelessness a bed, four walls, and basic services to live out the rest of their days, we found that PSH may also leave residents uncertain and fearful of dying alone, in pain, and without peace. If PSH programs do not address end-of-life directly, residents may delay seeking appropriate care at the end of life, remain uninformed about EOLC options, and die in needlessly unfortunate conditions. Providers and policymakers should be aware of the specific issues related to EOLC for people who live in PSH and realize that these may be different from individuals who are currently unhoused or receiving EOLC in a hospital or other facility. Permanent supportive housing staff and managers should also consider the types of services and programs that might address the end-of-life, harnessing the community strength within PSH to better support people who live their final days there.

## Appendix

### Abbreviations

ACP: advance care planning
EOLC: end-of-life care
PEH: people experiencing homelessness
PSH: permanent supportive housing
